# Serum proteomic analysis identifies sex-specific differences in lipid metabolism and inflammation profiles in adults diagnosed with Asperger syndrome

**DOI:** 10.1186/2040-2392-5-4

**Published:** 2014-01-27

**Authors:** Hannah Steeb, Jordan M Ramsey, Paul C Guest, Pawel Stocki, Jason D Cooper, Hassan Rahmoune, Erin Ingudomnukul, Bonnie Auyeung, Liliana Ruta, Simon Baron-Cohen, Sabine Bahn

**Affiliations:** 1 Department of Chemical Engineering & Biotechnology, University of Cambridge, Tennis Court Road, Cambridge, UK; 2 Autism Research Centre, Department of Psychiatry, University of Cambridge, 18B Trumpington Road, CB2 8AH, Cambridge, UK; 3 Division of Child Neurology and Psychiatry, Department of Developmental Neuroscience, Stella Maris Scientific Institute, Pisa, Italy; 4 Department of Neuroscience, Erasmus Medical Centre, Rotterdam, Netherlands

**Keywords:** Asperger Syndrome, Sex, Proteomics, Biomarkers, Lipid transport, Growth, Inflammation

## Abstract

**Background:**

The higher prevalence of Asperger Syndrome (AS) and other autism spectrum conditions in males has been known for many years. However, recent multiplex immunoassay profiling studies have shown that males and females with AS have distinct proteomic changes in serum.

**Methods:**

Here, we analysed sera from adults diagnosed with AS (males = 14, females = 16) and controls (males = 13, females = 16) not on medication at the time of sample collection, using a combination of multiplex immunoassay and shotgun label-free liquid chromatography mass spectrometry (LC-MS^E^). The main objective was to identify sex-specific serum protein changes associated with AS.

**Results:**

Multiplex immunoassay profiling led to identification of 16 proteins that were significantly altered in AS individuals in a sex-specific manner. Three of these proteins were altered in females (ADIPO, IgA, APOA1), seven were changed in males (BMP6, CTGF, ICAM1, IL-12p70, IL-16, TF, TNF-alpha) and six were changed in both sexes but in opposite directions (CHGA, EPO, IL-3, TENA, PAP, SHBG). Shotgun LC-MS^E^ profiling led to identification of 13 serum proteins which had significant sex-specific changes in the AS group and, of these, 12 were altered in females (APOC2, APOE, ARMC3, CLC4K, FETUB, GLCE, MRRP1, PTPA, RN149, TLE1, TRIPB, ZC3HE) and one protein was altered in males (RGPD4). The free androgen index in females with AS showed an increased ratio of 1.63 compared to controls.

**Conclusion:**

Taken together, the serum multiplex immunoassay and shotgun LC-MS^E^ profiling results indicate that adult females with AS had alterations in proteins involved mostly in lipid transport and metabolism pathways, while adult males with AS showed changes predominantly in inflammation signalling. These results provide further evidence that the search for biomarkers or novel drug targets in AS may require stratification into male and female subgroups, and could lead to the development of novel targeted treatment approaches.

## Background

Asperger syndrome (AS) is a subgroup within autism spectrum conditions (ASCs) [1]. The prevalence of AS in the general population is about 1% [2,3]. ASC leads to impairments in reciprocal social interaction and communication, alongside unusually restricted interests and repetitive behaviour [4], although people with AS develop language at the normal age and have no general cognitive delay. Similar to other forms of ASC, the prevalence of AS is higher in males compared to females with an approximate 4:1 ratio [5]. This suggests that sex-specific differences may affect its susceptibility, aetiology and/or manifestation.

In line with this, previous multiplex immunoassay profiling studies targeting specific classes of proteins have identified diagnosis-sex interactions in serum molecules such as cytokines, steroid and metabolic hormones, growth factors and lipid transport in adults with AS [6,7]. In these studies, females with AS had a higher number of changes in the levels of lipid and hormone-related molecules, and males with AS showed more changes in molecules related to dysfunction of immune or inflammatory pathways. However, individuals with AS often present with co-morbid psychiatric, neurological, gastrointestinal, metabolic, cardiovascular, gynaecological or musculoskeletal conditions [8]. This can make interpretation of the results of proteomic analyses difficult as the findings may be affected by drug- and lifestyle-related confounding factors.

Here, we attempt to minimize this possibility by analyzing samples from people with AS (n = 30) and controls (n = 29) who were not on medication at the time of blood collection and accounting for daily lifestyle routines. In addition, we used a combination of multiplex immunoassay and shotgun liquid chromatography mass (LC-MS^E^) profiling platforms to increase the analytical coverage to a wider range of protein classes [9]. The main objective was to identify sex-specific protein alterations in serum from people with AS compared to controls.

## Methods

### Clinical samples

Informed written consent was given by all participants. The protocols were approved by the UK National Health Service Cambridge Research Ethics Committee and studies were carried out in accordance with the Declaration of Helsinki. Recruitment of participants with AS was carried out as described by Schwarz *et al*. [6] and diagnoses were made by clinical psychologists or psychiatrists based on *Diagnostic and Statistical Manual of Mental Disorders IV-Text Review* (*DSM-IV-TR*)**.** All participants completed the Autism Spectrum Quotient (AQ) and the Empathy Quotient (EQ) forms [10]. Participants with a family history of serious mental illness or metabolic, cardiovascular or inflammatory diseases were excluded to minimise these as potential confounding factors. Samples from people with AS or controls who were not taking medications (antidepressants, antipsychotics, immunosuppressants, insulin) or using tobacco or marijuana at the time of sample collection were used in the current study to minimise the possibility of detecting drug-related proteomic changes (Table 1). Lifestyle information such as exercise level, alcohol intake, and oral contraception in females was also documented (Table 1).

**Table 1:** Demographics of non-medicated people with Asperger syndrome (AS) and controls used in the study

	**Male**		**Female**	
	**Patient**	**Control**	**Patient**	**Control**
**Sample number**	14	13	16	16
**Age (years)**	31 ± 9	31 ± 6	33 ± 9	34 ± 5
**BMI (kg/m**^**2**^**)**	24 ± 3	25 ± 4	26 ± 5	25 ± 6
**Smoking (yes/no)**	2/12	3/10	4/12	2/14
**Exercise level**	5/4/5/0/0	8/3/2/0/0	1/2/10/2/1	6/2/7/1/0
**Alcohol**	2/3/0/0/4/5	6/1/0/2/1/3	1/1/0/0/3/11	5/5/2/2/1/1
**Oral contraception**	NA	NA	7/9	4/12
**AQ score**	38 ± 13	15 ± 6	39 ± 13	14 ± 5
**EQ score**	28 ± 14	37 ± 14	15 ± 16	52 ± 10

Values are represented as mean ± standard deviation. BMI = body mass index. Exercise: high activity/moderate activity/low activity/sedentary/NA. Alcohol: 1to 5 units/6 to 10 units/11 to 15 units/16 to 20 units/none/NA. Oral contraception: never/past (not current).

### Sample collection

Blood samples were collected into 7.5 mL S-Monovette serum tubes (Sarstedt; Numbrecht, Germany) and placed at room temperature for two hours to allow coagulation, according to standard protocols. After this, the tubes were centrifuged at 1,100 × g for ten minutes to pellet the clotted material and other debris. The resulting serum supernatants were transferred into LoBind Eppendorf tubes (Hamburg, Germany) and stored at -80°C.

### Multiplex immunoassay analysis

Serum samples from drug-free people with AS (n = 30) and controls (n = 29) were analyzed using the HumanMAP panel comprised of immunoassays for 119 analytes (Additional file 1: Table S1) in a Clinical Laboratory Improved Amendments-certified laboratory at Myriad-RBM (Austin, TX, USA) as described previously [6,11]. The assays were calibrated using duplicate standard curves of each analyte and raw intensity measurements converted to protein concentrations using proprietary software. All measurements were conducted using randomized samples under blind conditions to minimize biases or batch effects.

### Mass spectrometry analysis

Depletion of abundant proteins was carried out in 40 μL of serum using the Human 14 Affinity Removal System (Agilent Technologies, Santa Rosa, CA, USA) on the AKTA purifier system (GE Healthcare, Uppsala, Sweden). This was carried out to increase the detection of higher numbers of low abundance proteins that are potentially masked by the more abundant serum components. The flow through fractions containing the low abundance proteins were exchanged into 50 mM ammonium bicarbonate (pH 8.0) using pre-washed 5 kDa-molecular weight cut-off Centricon tubes (Agilent Technologies; Santa Rosa, CA, USA). Protein concentrations were determined using the Biorad DC protein assay according to standard protocols (Hercules, CA, USA). In order to reduce disulfide bonds on proteins, samples were incubated for 30 minutes at 60°C with 100 mM dithiothreitol (Sigma Aldrich; Poole, UK). After this, 200 mM iodoacetamide (2.63 μL; Sigma Aldrich, Poole, UK) was added to each sample to alkylate the reduced cysteine residues, by incubation in the dark for 30 minutes at room temperature. Proteins were then digested using sequencing grade modified trypsin (Promega; Madison, WI, USA) at a ratio of 1:50 (w/w trypsin/protein) for 17 hours at 37°C. Digestions were stopped by addition of 1:60 8.8 M HCl to each sample. Samples were stored at -80°C prior to LC-MS^E^ analysis.

All solvents used for chromatography were of mass spectrometry grade (Fisher Scientific; Loughborough, UK). Buffers used were (A) 0.1% formic acid in water and (B) 0.1% formic acid in acetonitrile. Samples were diluted with buffer A to a final 0.12 μg/μL protein concentration and injected into the system. Each sample was analysed twice followed by alternating injections of a blank or a standard of 25 fmol/μLtryptically-digested yeast enolase (Waters Corporation; Milford, MA, USA). The samples were analysed on a nanoAquity ultra-performance liquid chromatography quadrupole time-of-flight (UPLCQTOF) Premier mass spectrometer (Waters Corporation, Elstree, UK) with a gradient starting at 97% buffer A (3% buffer B), followed by ramping to 70% A in 80 minutes, 70% to 5% in 10 minutes, running isocratically at 5% A for 10 minutes, then returning to initial conditions over 1 minute. The analytical column was coupled through a 10 μm fused-silica emitter (New Objective; Woburn, MA, USA) to the mass spectrometer, which was operated in positive V mode (resolution: 10,000 full width at half-maximum). The alternative scanning, data-independent expression mode (LC-MS^E^) was achieved with a setting for the low collision energy of 5 eV and the high collision energy ramped between 15 and 42 eV per scan. Acquisition time in each function was 0.6 seconds. Argon was used as the collision gas. Molecular ions were mass-corrected using the monoisotopic mass of the doubly-charged precursor of glufibrinopeptide B (785.8426 mass/charge), which was infused continuously using a reference spray apparatus.

The ProteinLynx Global Server (PLGS, version 2.4; Waters Corporation, Elstree, UK) was used for smoothing, centring, de-isotoping and charge state reduction of mass spectral peaks. Peptide fragment ions were allocated to peptide precursor ions based on identical retention times and elution profiles. For protein identification, an algorithm described by Li and colleagues was for searching the human Swiss-Prot database version 57.4 [12]. Time alignment was accomplished using the Elucidator™ software (Rosetta Biosoftware; Seattle, WA, USA) by applying the PeakTeller algorithm [13]. Peptide and fragment ion intensities were normalised to the total ion current and this required detection of these ions in both technical replicates of each sample and in at least 67% of the samples within each group. Protein intensities were calculated by summing the intensities of all peptide ions (mean values of technical replicates) associated with specific proteins.

### Statistical analysis

Principal component analysis (PCA) was carried out using the software SIMCA P+, v 2.12 (Umetrics; Stockholm, Sweden) to identify potential outliers in mass spectrometry data. PCA showed no clustering of the data based on demographic variables (data not shown). However, samples did show clustering based on analysis order. Therefore, protein intensities in mass spectrometry data were normalised using the median intensity of each batch to remove this effect. All other statistical tests were conducted using the free statistical software package R, v. 2.15.0 ( http://www.r-project.org). Multiplex immunoassay data were first pre-processed by removing analytes containing more than 30% missing values, resulting in 33 analytes being discarded and leaving 114 for analysis. The proportion of missing values in this dataset was less than 2%. Remaining missing values were imputed with twice the maximum or half the minimum analyte concentrations for measurements above and below the limits of quantitation, respectively. Missing values resulting from insufficient sample volume were replaced by the analyte mean. Both multiplex immunoassay and MS data were log_e_ transformed to stabilise variance and improve normality and outliers outside three standard deviations of the mean were removed. Differences in molecular levels between individuals with AS and control individuals were assessed for each analyte using stepwise regression, with sex, age, BMI, and exercise as additional covariates. *P*-values were adjusted to control the false discovery rate (FDR). Sex-diagnosis interactions were analysed in the same manner for each analyte. Those analytes with significant sex-diagnosis interactions (*P* < 0.05) were re-analysed for males and females separately and classified as being changed in females with AS (female-specific), changed in males with AS (male-specific), or in both with opposing directional changes (qualitative interaction). Analytes with changes of less than 10% were discarded. We also carried out Spearman correlation analyses in order to determine whether any of the measured serum analytes were correlated with AQ or EQ scores.

### Single reaction monitoring (SRM) mass spectrometry

Candidate proteins identified by label free LC-MS^E^ profiling were retested using single-reaction monitoring (SRM) on a XevoTQ-S mass spectrometer (Waters Corporation, Elstree, UK) coupled to a nanoAcquityUPLC system (Waters Corporation, Elstree, UK) as described previously [14]. This was aimed at providing a technical replication of the findings. Criteria for selecting candidate peptides representing the corresponding proteins for validation were based on peptide count, uniqueness and quality of transitions. Three peptides were selected for each target protein and isotopically-labelled peptides synthesised at JPT Peptide Technologies GmbH (Berlin, Germany). Data analyses were performed using the R-package SRM stats [15]. The settings used for group comparison were ‘restricted biological replication’ and ‘expanded technical replication’.

### *In silico* pathway analysis

The UniProt accession codes of proteins that showed diagnosis-sex interactions were uploaded into the Ingenuity Pathways Knowledge Database (IPKB; Ingenuity®™ Systems; Mountain View, CA, USA). The pathways most significant to the dataset were determined by automated overlay of the identified proteins onto predefined pathway maps in the IPKB. Fisher’s right-tailed exact test was used to calculate *P* values associated with the identified pathways. The significance of the association between the dataset and canonical pathways was measured by the ratio of the number of significant molecules divided by the total number of molecules in the canonical pathway and by the Fisher’s exact test *P* value.

## Results

### Multiplex immunoassay

Multiplex immunoassay profiling of serum samples resulted in identification of 16 analytes that were present at significantly different levels between drug-free individuals with AS (n = 30) and controls (n = 29) after adjustment for age, BMI, and exercise (Table 2). The analytes showing the largest ratiometric differences included neuronal cell adhesion molecule that was increased with a ratio of 1.4 in AS compared to controls, and fatty acid binding protein and growth hormone that were decreased with ratios less than 0.5.

**Table 2:** Identification of analytes altered between individuals with Asperger syndrome (AS) (n = 30) and controls (n = 29) using multiplex immunoassay analysis

	***P*-value**	**FDR**	**Ratio**
Neuronal cell adhesion molecule	0.022	0.19	1.40
IL-5	0.007	0.13	1.28
CD40	0.008	0.13	1.21
Cortisol	0.003	0.088	1.20
TNF-alpha	0.024	0.19	1.20
IL-7	0.012	0.15	1.18
BDNF	0.015	0.17	1.16
Sortilin	0.007	0.13	1.15
Serum glutamic-oxaloacetic transaminase	0.003	0.088	0.82
Apolipoprotein A1	0.018	0.19	0.79
Immunoglobulin M	0.002	0.088	0.73
HB-epidermal growth factor	0.002	0.088	0.70
Eotaxin-3	0.048	0.29	0.60
Ferritin	0.010	0.14	0.60
Fatty acid binding protein	0.030	0.20	0.50
Growth hormone	0.029	0.20	0.39

The results are adjusted for age and BMI. FDR = false discovery rate. Ratio = AS/control. Grey shading indicates these proteins had a significant sex-diagnosis interaction.

**Figure 1:**
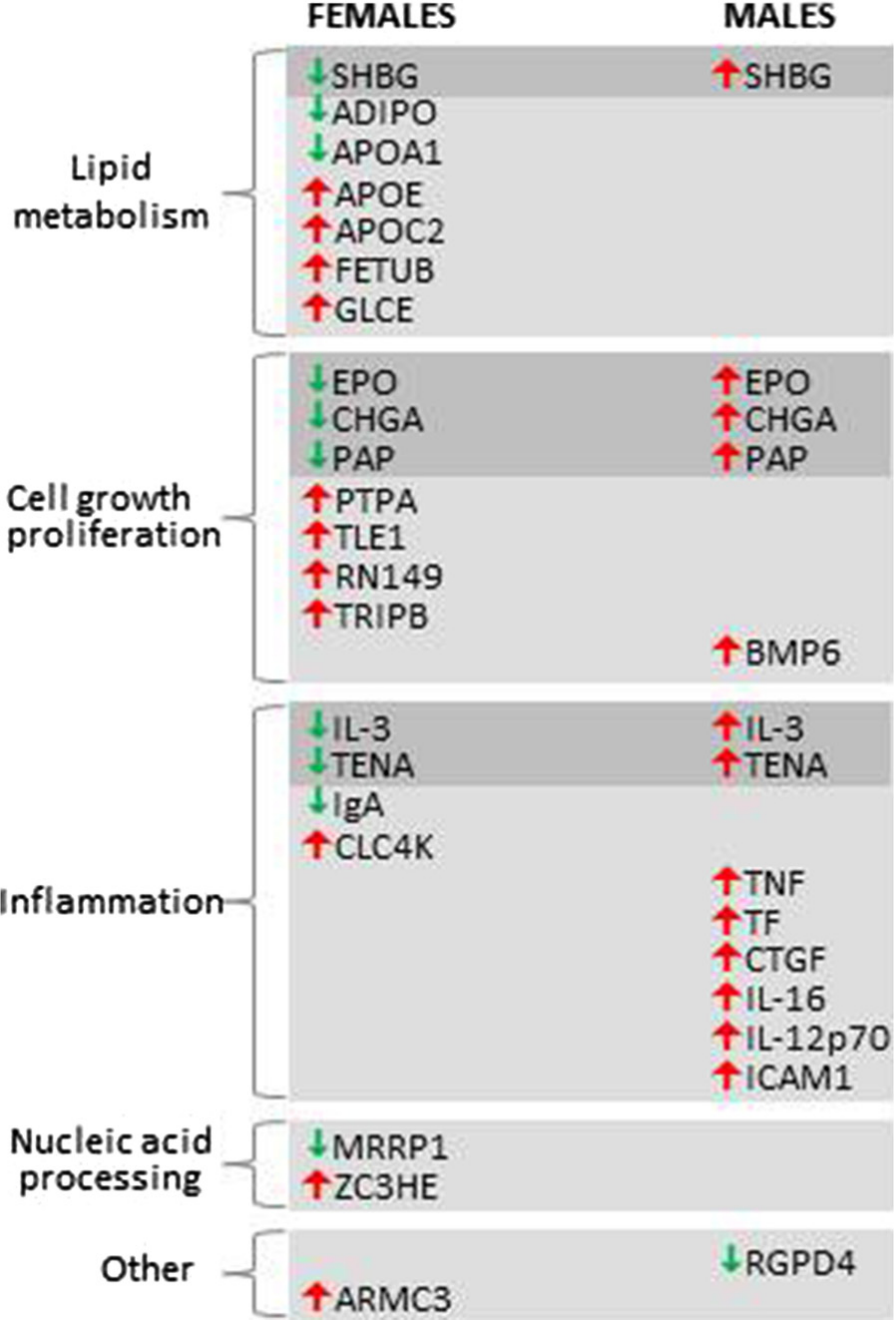
**Sex-specific and common changes in people with Asperger syndrome (AS) compared to controls.** The red arrows indicate an increase and the green arrows show a decrease of the protein in AS relative to controls. The proteins in grey boxes are changed in both males in females but in opposite directions. The abbreviations are as indicated in Table 2.

We then identified 16 serum proteins changed in a sex-specific manner in AS. Seven proteins (BMP6, TNF, TF, CTGF, IL-16, IL-12p70, ICAM-1) were increased specifically between males with AS (n = 14) and male controls (n = 13) and three proteins (ADIPO, IgA, APOA1) were decreased in females with AS (n = 16) in comparison to female controls (n = 16) (Figure 1 and Table 3). In addition, six proteins (CHGA, TENA, SHBG, PAP, EPO, IL-3) showed opposite-increased or -decreased concentrations between the AS male and AS female groups. In the latter case, the differences for SHBG (*P* = 0.065) and EPO (*P* = 0.060) did not reach significance between females with AS and female controls (Figure 1 and Table 3). BMP6 showed the highest male-specific increase in AS compared to controls at a ratio of 3.04 and IL-3 showed the strongest decrease with a ratio of 0.28. Conversely, IL-3 showed the highest female-specific increase with a ratio of 1.87 and APOA1 showed the greatest decrease at a ratio of 0.63 (Table 3).

**Table 3:** Summary of significant sex x diagnosis interactions of serum molecules measured by multiplex immunoassay

	**Interaction**		**Males**		**Females**	
**Protein**	***P*-value**	**q-value**	**Ratio**	***P*-value**	**Ratio**	***P*-value**
Bone morphogenic protein-6 (BMP6)	0.023	0.193	**3.04**	**0.001**	0.85	0.703
*Tumour necrosis factor-alpha (TNF)*	0.017	0.191	**1.45**	**0.003**	1.02	0.192
*Tissue factor (TF)*	0.012	0.154	**1.39**	**0.001**	0.73	0.151
*Connective tissue growth factor (CTGF)*	0.001	0.037	**1.29**	**0.001**	0.83	0.091
Interleukin-16 (IL-16)	0.025	0.196	**1.17**	**0.008**	0.88	0.134
*Interleukin-12p70 (IL-12p70)*	0.005	0.070	**1.16**	**0.008**	0.91	0.074
*Intracellular adhesion molecule-1 (ICAM1)*	0.027	0.196	**1.15**	**0.010**	1.00	0.343
*Chromogranin A (CHGA)*	<0.001	0.008	**1.70**	**0.001**	**0.77**	**0.019**
*Tenascin C (TENA)*	0.002	0.037	**1.28**	**0.049**	**0.66**	**0.007**
*Sex hormone binding globulin (SHBG)*	0.026	0.191	**1.20**	**0.036**	0.76	0.065
Prostatic acid phosphatase (PAP)	0.004	0.058	**1.19**	**0.040**	**0.82**	**0.028**
*Erythropoietin (EPO)*	0.044	0.282	**0.49**	**0.021**	1.36	0.060
*Interleukin-3 (IL-3)*	<0.001	0.022	**0.28**	**0.006**	**1.87**	**0.039**
Adiponectin (ADIPO)	0.037	0.251	1.19	0.453	**0.77**	**0.037**
Immunoglobulin A (IgA)	0.022	0.192	1.05	0.852	**0.73**	**0.006**
*Apolipoprotein A1 (APOA1)*	0.002	0.037	1.05	0.857	**0.63**	**0.001**

Indicated are the *P*-values and FDR (false discovery rate) of the sex-diagnosis interactions and the separate ratio (AS/control; calculated using geometric means) changes with *P*-values in males in females. Values in bold font indicate significant sex differences in individuals with AS and typical individuals. Analytes in italic style font were identified in our previous study of AS individuals, which did not account for potential drug effects [6]. In the overlapping region, changes in molecular levels are in opposite directions in males and females.

We found that the levels of SHBG were decreased in females with AS compared to both males with AS and controls, which could be associated with higher levels of free testosterone. We estimated the free testosterone levels in both males and females by dividing total testosterone (measured by the multiplex immunoassay panel) by the SHBG levels. This is termed the free androgen index (FAI). In females, the FAI showed an increased ratio of 1.63 (*P* = 0.0275) in individuals with AS compared to controls. In males, the FAI was found at a ratio of 0.85 in AS compared to controls although this was not significant (*P* = 0.2206). Given the prior association of increased testosterone-related medical conditions in adult females with ASC [16], we tested the levels of testosterone, SHBG and the FAI in relation to AQ and the EQ scores in AS using Spearman correlation analysis. However, this revealed no significant correlations.

### Mass spectrometry

LC-MS^E^ proteome profiling of serum was performed to identify novel gender-specific serum biomarkers not analysed using the multiplex immunoassay platform. Using LC-MS^E^ we measured the levels of 9,068 serum peptides, which corresponded to 313 proteins using the criteria outlined in the methods section. We found 13 proteins with significant sex-diagnosis interactions, 12 of which (ARMC3, PTPA, TLE1, CLC4K, GLCE, APOC2, ZC3HE, FETUB, RN149, TRIPB, APOE, MRRP1) were altered specifically in females with AS compared to female controls. Only one protein (RGPD4) was altered only in males with AS compared to male controls (Figure 1, Table 4).

**Table 4:** **Summary of significant sex-diagnosis interactions of serum molecules measured by LC-MS**^**E**^**profiling**

				**Males**		**Females**	
**Code**	**Protein**	**Interaction**	**FDR**	**Ratio**	***P*-value**	**Ratio**	***P*-value**
RGPD4	RANBP2-like and GRIP domain containing 5	0.015	0.723	**0.89**	**0.016**	1.07	0.893
ARMC3	Armadillo repeat containing 3	0.002	0.291	0.88	0.183	**1.29**	**0.001**
PTPA	PP 2A activator, reg subunit 4	0.038	0.723	0.97	0.681	**1.23**	**0.001**
TLE1	Transducin-like enhancer of split 1	0.049	0.723	0.90	0.394	**1.22**	**0.026**
CLC4K	CD207 molecule, langerin	0.017	0.723	0.96	0.720	**1.20**	**<0.001**
GLCE	Glucuronic acid epimerase	0.017	0.723	0.88	0.231	**1.19**	**0.018**
APOC2	Apolipoprotein C2	0.005	0.522	0.92	0.254	**1.19**	**0.001**
ZC3HE	Zinc finger CCCH-type containing 14	0.039	0.723	0.97	0.383	**1.16**	**0.020**
FETUB	Fetuin B	0.035	0.723	0.96	0.572	**1.15**	**0.004**
RN149	Ring finger protein 149	0.044	0.723	1.02	0.588	**1.14**	**0.042**
TRIPB	Thyroid hormone receptor interactor 11	0.012	0.723	0.94	0.610	**1.13**	**0.027**
APOE	Apolipoprotein E	0.030	0.723	0.99	0.851	**1.11**	**<0.001**
MRRP1	RNA G9 methyltransferase domain cont 1	0.044	0.723	1.06	0.592	**0.78**	**0.035**

Indicated are the *P*-values and FDR (false discovery rate) of the interactions and the separate ratio (AS/control; calculated using geometric means) changes with *P*-values in males in females. Values in bold font indicate significant differences between AS and typical individuals by sex.

**Figure 2:**
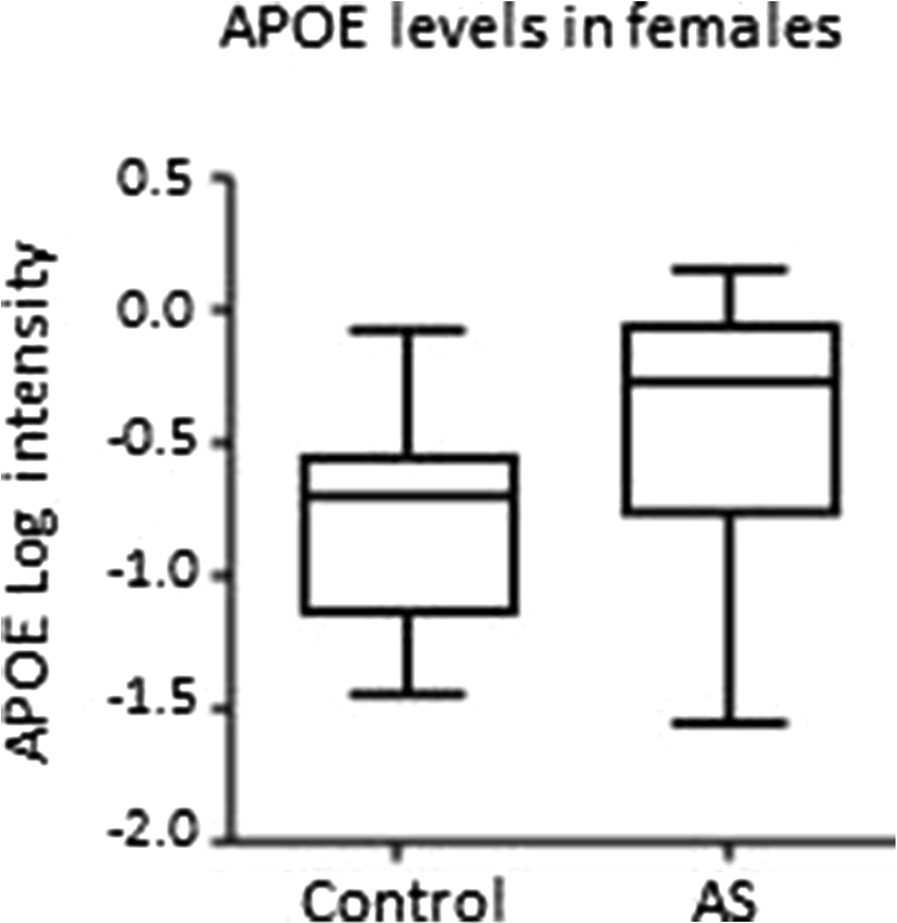
Validation of changes in apolipoprotein E levels in females with Asperger syndrome (AS) (n = 16) compared to female controls (n = 16) using Selective Reaction Monitoring (SRM) mass spectrometry.

ARMC3 and PTPA showed the highest increases in females with AS compared to female controls at ratios of 1.29 and 1.23, respectively. MRRP1 showed the greatest decrease in females with AS compared to female controls at a ratio of 0.78. The finding of increased APOE levels in females with AS compared to female controls was the most significant result in this study (*P* = 0.0002). Because the FDR for APOE was 0.72, we attempted to validate the findings using an orthogonal analysis as described in the methods section. An SRM mass spectrometry assay was established for APOE and this confirmed that it was increased in females with AS compared to female controls with a ratio change of 1.27 (*P* = 2.13E^-13^), which was more robust than the findings for the LC-MS^E^ study (1.11 fold) (Figure 2).

### *In silico* pathway analysis

The Uniprot accession codes for 19 proteins associated with females with AS were uploaded into the IPKB to identify the most significant networks, diseases and canonical pathways associated with the dataset. Note that no code for IgA was uploaded as this was not present in the database. A single network was identified which showed interactions for nine of these proteins (ADIPO, APOA1, APOC2, APOE, EPO, IL-3, PAP, SHBG, TENA) and the predominant function associated with these proteins was lipid metabolism. The most significant disease was listed as ‘cancer’ , although this was due to the effects on cell proliferation (*P* = 5.9E-06 - 5.0E-02), accounted for by nine proteins (ADIPO, APOA1, APOE, ARMC3, CHGA, EPO, IL-3, PAP, TENA). The most significant canonical pathway was FXR/RXRsignalling (1.9E-06) which was covered by four proteins (APOA1, APOC2, APOE, FETUB). Of the 13 male AS-associated proteins, nine of these (BMP6, CTGF, EPO, ICAM1, IL-3, IL-16, SHBG, TENA, TNF) were associated with a single network with a predominant function of cell to cell signalling. The top disease was inflammation signalling (*P* = 2.0E-06 - 4.2E-03; seven proteins: EPO, ICAM1, IL-3, IL-12p70, IL-16, TENA, TNF) and the top canonical pathway was differential regulation of cytokine production (*P* = 3.8E-07; three proteins: IL-3, IL-12p70, TNF).

## Discussion

This is the first molecular profiling study using a combination of multiplex immunoassay and mass spectrometry to investigate sex-specific differences in serum from adults with AS compared to typical controls. All participants were drug-free at the time of sample collection. Specifically, samples were not analysed from individuals taking medications or substances such as antidepressants, antipsychotics, immunosuppressants, antidiabetics, tobacco or marijuana. This study design increases the chances that the identified findings are linked to the underlying pathways altered in AS and minimises possible confounding effects of drug treatment. Analysis of the combined cohort led to identification of 16 analytes that were present at significantly different levels in AS compared to controls. Several of these molecules have been implicated previously in ASC, such as neuronal cell adhesion molecule [17], cortisol [18], TNF-alpha [19], brain-derived neurotrophic factor [20], IL-5 [21], eotaxin-3 [22], immunoglobulin M [23], ferritin [24] and growth hormone [25]. In the next phase of the study, serum samples from approximately equal numbers of males and females were analysed to allow determination of sex-specific changes. This resulted in identification of 29 proteins with significant sex-diagnosis interactions. Fifteen of these (ADIPO, APOA1, APOC2, APOE, ARMC3, CLC4K, FETUB, GLCE, IgA, MRRP1, PTPA, RN149, TLE1, TRIPB, ZC3HE) showed altered levels specifically in female patients, eight (BMP6, CTGF, IL-16, IL-12p70, ICAM-1, RGPD4, TF, TNF) were altered in males, and six (CHGA, TENA, SHBG, PAP, EPO, IL-3) showed opposite changes in females and males. For the multiplex immunoassay analysis, the changes in 11 proteins (APOA1, CHGA, CTGF, EPO, IL-3, IL-12p70, ICAM1, SHBG, TENA, TF and TNF) were consistent with those found in our previous study [6]. This suggests that the sex-specific alterations in these molecules were not influenced by drug effects.

*In silico* pathway analysis revealed that the predominant pathway affected in females with AS was lipid metabolism. This is in line with previous studies showing alterations in circulating lipids such as cholesterol in individuals with ASC [26,27]. All steroids are synthesized from cholesterol and, in the brain, these are involved in regulation of neuronal processes such as GABA_A_ and NMDA receptor signalling, myelin formation and synaptogenesis [28]. This may be of relevance to the current findings since diseases marked by impaired cholesterol biosynthesis, such as Smith-Lemli-Opitz Syndrome (SLOS), are associated with an increased incidence of ASC [29]. Furthermore, treatment of SLOS patients with cholesterol leads to fewer autistic behaviours, infections, and symptoms of irritability and hyperactivity, with improvements in physical growth, sleep and social interactions. Consistent with potential effects on lipid metabolism, we found that females with AS had altered levels of three members of the apolipoprotein family (APOA1, APOC2, APOE), which are involved in cholesterol transport. There is extensive evidence that the levels of some apolipoproteins are disturbed in ASC and other conditions [28]. The present study suggests these effects may be more prominent in females with ASC. This is also in line with other studies which have shown that APOA1 levels are reduced in women with polycystic ovary syndrome (PCOS), which has an increased prevalence in ASC relative to the general population [30].

We also found female-specific changes in ADIPO, GLCE, FETUB and SHBG, which all have functions related to lipid biosynthesis or metabolism [31-34]. In addition to its role in lipid-related pathways, SHBG also serves as the main transport protein for sex steroids such as oestrogen and testosterone [35]. According to the foetal androgen theory, high levels of testosterone and other steroid androgens during prenatal brain development can contribute to the development of ASC [16]. Previous studies have demonstrated that there is a correlation between high foetal testosterone concentrations with evidence of more masculinisedbehaviour in later life [16] and an increased number of autistic traits in the normal population [36]. Our finding of elevated levels of SHBG in females may indicate higher levels of free testosterone. This is of potential interest as several studies have found that administration of drugs such as statins can reduce the effects of hyperandrogenemia in conditions such as PCOS that are associated with high testosterone levels [37-39]. One report has hypothesized that statins may be a potential novel therapy in autism and epilepsy [40]. In addition, insulin-sensitizing agents appear to be efficacious in reducing testosterone levels and alleviating other symptoms of PCOS [41]. If such treatments are effective for ASC, the present results suggest that it may be more appropriate to apply these specifically in the case of females with AS. However, caution is needed in extrapolating from the current results as these are derived from adults and we do not know if these would hold for younger individuals. Nor do we make claims for treatment implications as side-effects have yet not been fully evaluated.

Most of the remaining proteins changes specifically in females identified by the combined proteomic profiling platforms are involved in regulation of cell growth, differentiation, survival or apoptosis [42-48]. This included changes in CHGA, EPO, PAP, PTPA, TLE1, RN149 and TRIPB. The others have roles in immune system function (IgA, IL-3, TENA, CLC4K [49,50]), regulation of brain-specific mRNAs (ZC3HE [51]) transfer RNA processing (MRRP1 [52]) or cell adhesion and mobility (ARMC3 [53]). Previous studies have shown a reduction of IgA levels in subgroups of people with ASC although the study design did not test for sex differences [49].

The majority of proteins that showed differences in males with AS specifically were associated with inflammation pathways. These included TNF-alpha, which has previously been identified in children with ASC, although not in a sex specific manner [54]. Effects of inflammation are identified frequently in studies of ASC and this could be due to the high proportion of males that are normally analysed in these studies. For example, previous studies found that ICAM-1 was decreased in autism [55], although we showed that it was increased significantly in adult males with AS. Other inflammation-related proteins that were found to be altered specifically in males with AS were TF, CTGF, IL-16 and IL-12p70 [56-58]. The finding of an inflammatory signature in males may have potential applications for a stratified medical approach. For example, males with AS exhibiting immune dysfunction might be candidates for treatment with anti-inflammatory drugs, subject to normal safety checks. Recently, an open label pilot study showed that treatment with a combination of the flavonoids luteolin and quercetin seemed to be effective in reducing autistic symptoms in children, with no major adverse effects [59]. In addition, a randomised double-blind placebo-controlled trial showed that a combination of risperidone and celecoxib was superior to risperidone alone in treating irritability, social withdrawal, and stereotypy of children with autism [60].

We also found that two proteins associated with other pathways were altered specifically in males with AS. These were BMP6 and RGPD4. BMP6 has been associated previously with proliferation and differentiation of cells [61] and RGPD4 belongs to family of proteins involved in intracellular trafficking and sorting [62]. Finally, six proteins (CHGA, TENA, SHBG, PAP, EPO and IL-3) were changed in both males and females with AS, although these changes occurred in opposite directions. Taken together, these findings provide further evidence for marked differences in the underlying affected pathways between males and females with AS.

There are several limitations to this study. First, there is a potential bias in the molecular classes of the investigated molecules. This is based on the proteins targeted by the multiplexed immunoassay and mass spectrometry platforms that do not cover all functional classes of proteins. Therefore, it is possible that analysis of a different selection of molecules would lead to different conclusions from those drawn in this study. Another limiting factor was the small number of clinical serum samples tested. This was due to the rarity of such samples that could be obtained using strict standard operating procedures from both individuals with AS and matched controls. Also, the fact that this study included only AS individuals who were not on medication could result in a selection bias. For example, this could mean that samples from the less severe cases were tested, such as those without associated anxiety or depression. In addition, as mentioned earlier, the current study has only investigated adults so cannot account for age-related differences that are likely to be important in ASC.

## Conclusion

In conclusion, we have identified sex-specific proteomic changes in sera from adults with AS. Females showed changes in proteins mainly associated with lipid transport and metabolism, including FAI, and males showed changes predominantly in inflammation pathways. Further exploration is warranted into the mechanisms by which these sexually dimorphic molecular phenotypes in AS arise. This may lead to deeper insights into the well-established sex differences in the clinical manifestation [63] and brain structure [64] and course of ASC. This may have implications for the development of novel targeted treatment approaches for improved outcomes, and for understanding sex-linked aetiological factors in autism [65].

## Abbreviations

ADIPO: Adiponectin; ANOVA: Analysis of variance; APO: Apolipoprotein; AQ: Autism Spectrum Quotient; ARMC3: Armadillo repeat containing 3; AS: Asperger syndrome; ASC: Autism spectrum condition; BMI: Body mass index; BMP6: Bone morphogenic protein6; CHGA: Chromogranin A; CTGF: Connective tissue growth factor; DSM-IV-TR: The *Diagnostic and Statistical Manual of Mental Disorders IV-Text Review*; EPO: Erythropoietin; FAI: Free androgen index; FDR: False discovery rate; FETUB: Fetuin B; GABA: Gamma aminobutyric acid; GLCE: Glucuronic acid epimerase; ICAM-1: Intracellular adhesion molecule-1; Ig: Immunoglobulin; IL: Interleukin; IPKB: Ingenuity Pathways Knowledge Database; kDa: KiloDaltons; LC-MSE: Liquid chromatography-mass spectrometry; MAP: Multi-Analyte Profiling; MRRP1: RNA G9 methyltransferase domain cont 1; NMDA: N-methyl-D-aspartate; PAP: Prostatic antigen phosphate; PCA: Principle component analysis; PCOS: Polycystic ovary syndrome; PTPA: Protein phosphatase 2A activator; QTOF: Quadrupole time-of-flight; RGPD4: RANBP2-like and GRIP domain containing 5; RN149: Ring finger protein 149; SHBG: Sex hormone binding globulin; SLOS: Smith-Lemli-Opitz Syndrome; SRM: Selective Reaction Monitoring; STARD: Standards for Reporting of Diagnostic Accuracy; TENA: Tenascin C; TLE1: Transducin-like enhancer of split 1; TNF: Tumour necrosis factor; TRIPB: Thyroid hormone receptor interactor 11; UPLC: Ultra-performance liquid chromatography; ZC3HE: Zinc finger CCCH-type containing 14.

## Competing interests

SB and JDC are consultants for Myriad-RBM. This does not affect policies regarding sharing of data and materials specified by this journal.

## Authors’ contributions

HS, JMR, PS and JDC carried out the molecular profiling data analyses. PCG, SB and SB-C interpreted the results, prepared the figures and tables, and wrote the manuscript. LR, SB, HR and SB-C designed the clinical studies and edited the manuscript. EI, LR, and BA coordinated clinical data collection. SB and SB-C conceived the study, interpreted the results and edited the manuscript. All authors read and approved the final manuscript.

## Authors’ information

Simon Baron-Cohen and Sabine Bahn are senior authors.

## Supplementary Material

Additional file 1 Analytes measured using multiplex immunoassay platform. Click here for file
