# Supplementary material for: Serum proteomic analysis identifies sex-specific differences in lipid metabolism and inflammation profiles in adults diagnosed with Asperger syndrome
Source: Mol Autism. 2014 Jan 27;5:4. doi: 10.1186/2040-2392-5-4 (PMC3905921; doi:10.1186/2040-2392-5-4)
Supplement: Additional file 1 — Analytes measured using multiplex immunoassay platform. [file 2040-2392-5-4-S1.doc]

**Supplementary Table S1. Analytes measured using multiplex immunoassay platform**

Alpha-1 Antitrypsin

ACE (Angiotensin Converting Enzyme)

Adiponectin

Alpha-2 Macroglobulin

Alpha-Fetoprotein

ANG-2 (Angiopoietin 2)

Angiotensinogen

Apolipoprotein A1

Apolipoprotein CIII

Apolipoprotein H

AXL

Beta-2 Microglobulin

BLC (B-Lymphocyte Chemoattractant)

BMP-6

BDNF (Brain-Derived Neurotrophic Factor)

Complement 3

Cancer Antigen 125

Cancer Antigen 19-9

CD40

CD40 Ligand

Carcinoembryonic Antigen

CgA (Chromogranin A)

Creatine Kinase-MB

Cortisol

C Reactive Protein

CTGF (Connective Tissue Growth Factor)

EGF

EGF-R

ENA-78

Endothelin-1

EN-RAGE

Eotaxin

Eotaxin-3

Erythropoietin

FABP

Factor VII Fas

Fas-Ligand

Ferritin

FGF basic

Fibrinogen

FSH (Follicle-Stimulating Hormone)

G-CSF

Growth Hormone

GRO-alpha

GST

Haptoglobin

HB-EGF

HCC-4

HGF (Hepatocyte growth factor)

I-309

ICAM-1

IFN-gamma

IgA

IgE

IGF BP-2

IGF-1

IgM

IL-10

IL-12p40

IL-12p70

IL-13

IL-15

IL-16

IL-17

IL-18

IL-1beta

IL-1ra

IL-3

IL-4

IL-5

IL-7

IL-8

Insulin

Leptin

LH (Luteinizing Hormone)

Lipoprotein (a)

MCP-1

MDC

MIF

MIP-1alpha

MIP-1beta

MMP-2

MMP-3

MMP-9

Myeloperoxidase

Myoglobin

NrCAM

PAI-1

Pancreatic Polypeptide

Prostatic Acid Phosphatase

PDGF

Progesterone

Prolactin

Prostate Specific Antigen, Free

PARC

RANTES

Resistin

Serum Amyloid P

Stem Cell Factor

SGOT

SHBG

SOD

Sortilin

sRAGE

Thyroxine Binding Globulin

Tenascin C

Testosterone

Tissue Factor

TIMP-1

TNF RII

TNF-alpha

TNF-beta

Thrombopoietin

TRAIL-R3

Thyroid Stimulating Hormone

TSP-1

VCAM-1

VEGF

von Willebrand Factor
